# Joint analysis of WES and RNA‐Seq identify signature genes related to metastasis in prostate cancer

**DOI:** 10.1111/jcmm.17781

**Published:** 2023-06-28

**Authors:** Chongjun Xiang, Yue Li, Wenting Wang, Huiying Tao, Ning Liang, Shuang Wu, Tianxi Yu, Xin Cui, Yaqi Xie, Hongwei Zuo, Chunhua Lin, Fuyi Xu

**Affiliations:** ^1^ The 2nd Medical College of Binzhou Medical University Yantai China; ^2^ Department of Urology the Affiliated Yantai Yuhuangding Hospital of Qingdao University Yantai China; ^3^ Department of Central Laboratory the Affiliated Yantai Yuhuangding Hospital of Qingdao University Yantai China; ^4^ School of Clinical Medicine Weifang Medical University Weifang China; ^5^ Shandong Technology Innovation Center of Molecular Targeting and Intelligent Diagnosis and Treatment, School of Pharmacy Binzhou Medical University Yantai China

**Keywords:** gene expression, metastasis, prostate cancer, RNA‐Seq, WES

## Abstract

Prostate cancer (PCa) has a certain degree of heritability, and metastasis occurs as cancer progresses. However, its underlying mechanism remains largely unknown. We sequenced four cases of cancer without metastasis, four metastatic cancer, and four benign hyperplasia tissues as controls. A total of 1839 damaging mutations were identified. Pathway analysis, gene clustering, and weighted gene co‐expression network analysis were employed to find characteristics associated with metastasis. Chr19 had the most mutation density and 1p36 had the highest mutation frequency across the genome. These mutations occurred in 1630 genes, including the most frequently mutated genes *TTN* and *PLEC*, and dozens of metastasis‐related genes, such as *FOXA1*, *NCOA1*, *CD34*, and *BRCA2*. Ras signalling and arachidonic acid metabolism were uniquely enriched in metastatic cancer. Gene programmes 10 and 11 showed the signatures indicating the occurrence of metastasis better. A module (135 genes) was specifically associated with metastasis. Of them, 67.41% reoccurred in program 10, with 26 genes further retained as the signature genes related to PCa metastasis, including *AGR3*, *RAPH1*, *SOX14*, *DPEP1*, and *UBL4A*. Our study provides new molecular perspectives on PCa metastasis. The signature genes and pathways could be served as potential therapeutic targets for metastasis or cancer progression.

## 1 INTRODUCTION

With nearly 1.4 million new cases and 375,000 deaths worldwide, prostate cancer (PCa) is the second most frequent cancer, the fifth leading cause of cancer death among men, and the fourth incidence of new cancer cases among both sexes in 2020.^1^ PCa shows extensive intra‐ and inter‐tumour heterogeneity and diverse clinical behaviour, with some patients dying of metastasis within 2–3 years after diagnosis while others can live for more than 10 years, indicating the potential role of genomic diversity.^2^

Tumour metastasis is a process by which cancer cells disperse from a primary site to distant organs or tissues. In addition to sharing some key driver mutations with the primary tumour from which they arise, metastatic tumours often develop additional mutations as they evolve during metastasis and treatment.^3^, ^4^ With the cancer progress, PCa tends to metastasize, especially bone. Studies have identified several genes or pathways implicated in metastasis. For instance, *PMEPA1* induced by *TGFβ1* was identified to suppress PCa metastasis to the bone by blocking TGF‐β signalling.^5^ *MAOA* was demonstrated to involve in the EMT process^6^ and promote bone metastasis through activation of paracrine Shh signalling in osteoblasts.^7^ Activated *RANK–RANKL* signalling in PCa cells is implicated in colonizing cancer cells in the bone.^8^ The Wnt signalling pathway also plays a role in PCa cells by promoting osteoblast differentiation.^9^ Moreover, alterations in androgen signalling, DNA repair, and phosphoinositide 3‐kinase (PI3K) signalling, as well as recurrent mutations in some genes such as *FOXA1* and *IDH1*, can be frequently found in PCa metastasis.^10^, ^11^, ^12^, ^13^, ^14^

In recent years, next‐generation sequencing‐based analysis has greatly improved our understanding of the genomic definition and molecular characteristics of cancer. Large cohorts like The Cancer Genome Atlas (TCGA), Chinese Prostate Cancer Genome and Epigenome Atlas,^10^ and MET500^4^ have profiled the molecular signatures and provided insights into the PCa. However, systemically characterizing the PCa metastasis with a multi‐omics approach was merely reported.

In this study, PCa and PCa metastasis samples were deployed for whole exome sequencing (WES) and transcriptome sequencing. Both genomic and transcriptomic landscapes for PCa metastasis were characterized. More importantly, signature genes related to PCa metastasis were further identified.

## 2 MATERIALS AND METHODS

### 2.1 Clinical specimens and ethics statement

Twelve tissues were obtained from Yuhuangding Hospital, including four prostatic hyperplasia samples, four PCa samples, and four PCas with multiple metastases. All tissues were collected at the time of puncturing. Pathological diagnoses were conducted by two independent and expert pathologists. All tumour tissues were confirmed as prostate adenocarcinoma. The specimens were stored at −80°C until sequencing. None of the cancer patients received any therapy before their diagnosis. The Yuhuangding Hospital Ethics Committee approved the study protocol. All experiments complied with the relevant regulations, and all patients provided written informed consent.

### 2.2 Whole exome sequencing

Total genomic DNA was extracted and mechanically fragmented (3 μg, based on Qubit quantification) on the Covaris S220 Focused‐ultrasonicator (Covaris). Quality control was performed using the Agilent Bioanalyzer 2100 (Agilent Technologies) to ensure an average fragment size of 150–200 bp. End repair, A‐tailing, adaptor ligation, purification, and enrichment of DNA fragments were then performed. A 225‐ to 275‐bp band was selected, and exome capture was performed using the SureSelectXT Reagent Kit (Agilent Technologies). The DNA library was quantified using the Qubit Fluorometer (Life Technologies) and Agilent 2100 Bioanalyzer (Agilent Technologies). Samples were subjected to paired‐end sequencing using the Illumina NextSeq 500 platform with a 150‐bp read length. Fastp (v0.19.5)^15^ was used for quality control and the Burrows‐Wheeler Alignment tool (v0.7.12)^16^ was used to map the clean reads onto the human reference genome (GRCh37). Single nucleotide polymorphisms (SNPs) and Insertions and deletions (InDels) were called using SAMtools (v1.3.1)^17^ and BCFtools (v1.3.1).^18^ Annovar^19^ was used to annotate and predict the effects of the variants on genes.

### 2.3 Variants filtration

The variants were filtered with the following criteria: (1) variants classified as stop‐gain or stop‐loss, (2) variants classified as frameshift and nonsynonymous in cancer samples, (3) variants with minor allele frequency less than 1% in the human population (1000G, gnomAD and esp6500), (4) variants predicted to be deleterious in at least three of five in silicon analysis (Mutation Taster, PROVEAN, Polyphen2, SIFT, and CADD), of which the criterion for CADD was greater than 20.

### 2.4 RNA sequencing and quantification

Total RNA was extracted using the TRIzol reagent according to the manufacturer's protocol. RNA purity and quantification were evaluated using the NanoDrop 2000 spectrophotometer (Thermo Scientific). RNA integrity was assessed using the Agilent 2100 Bioanalyzer (Agilent Technologies). Then the libraries were constructed using TruSeq Stranded mRNA L.T. Sample Prep Kit (Illumina) according to the manufacturer's instructions. The libraries were sequenced on an Illumina HiSeq X Ten platform by O.E. Biotech Co., Ltd., and 150 bp paired‐end reads were generated.

Raw reads were first processed using Trimmomatic (v0.36),^20^ and the low‐quality reads were removed to obtain clean data. About 45M clean reads for each sample were retained. The clean reads were then mapped to the human genome (GRCh38) using HISAT2 (v2.2.1.0).^21^ The read count of each gene was obtained by HTSeq‐count (v0.9.1),^22^ and the fragments per kilobase of transcript per million fragments mapped (FPKM)^23^ of each gene was calculated using Cufflinks.^24^

### 2.5 Principal component analysis (PCA) and identification of differentially expressed genes (DEGs)

The factoextra package in R (v4.1.2) was used to complete PCA. The DESeq2 (v1.34.0)^25^ package in R (v4.1.2) was used to identify the DEGs. The negative binomial distribution was used for the raw read count matrix. DEGs demonstrated according to the adjusted *p* value <0.05 and |log2FC| > 1.

### 2.6 Clustering analysis

Genes were divided into 11 clusters with K‐means in R (v4.1.2). The average expression of each gene (FPKM value) in each group was calculated. The Gene cluster trend module in Hiplot (https://hiplot‐academic.com/basic/gene‐trend) was used to classify the different expression patterns.

### 2.7 Functional enrichment analysis

Webgestalt (http://www.webgestalt.org/)^26^ was used to analyze the molecular and functional characteristics of the DEGs. The resulting False Discovery Rate (FDR) lower than 0.05 was used to define the overrepresented KEGG pathways.

### 2.8 Weighted gene co‐expression analysis

We first removed genes whose expression value (FPKM) is equal to 0 in all samples. The filtered data was log‐transformed with log2(*x* + 1) and used as input for the Weighted Gene Co‐expression Network Analysis (WGCNA).^27^ The unsigned soft threshold power β of 10 was determined according to the standard scale‐free network. Then an unsigned co‐expression network was constructed based on the Topological Overlap Matrix and Hierarchical clustering algorithm. For each module, the summary profile (eigengene) was defined as the first principal component of the given module. The correlations between the eigengenes and the external traits were used for evaluating the module‐trait relationships.

## 3 RESULTS

WES and RNA‐Seq were performed to profile gene mutation and expression from four prostatic hyperplasia patients (as control group P), four PCa patients (PC group), and four PCas with multiple metastases (M_PC group). None of the cancer patients received any therapy before their diagnosis. We generated over 1.22 billion raw reads and an average of 100 million per sample from WES. The coverage of the sequencing depth ranged from 82.42 to 113.20X, with an average depth of 96.24X. We acquired 556.92 million raw reads from RNA sequencing in total and an average of 46.41 million for each sample. The WES and RNA sequencing statistics are summarized in Table S1.

### 3.1 Damaging mutations across the PCa and metastasis

**FIGURE 1:**
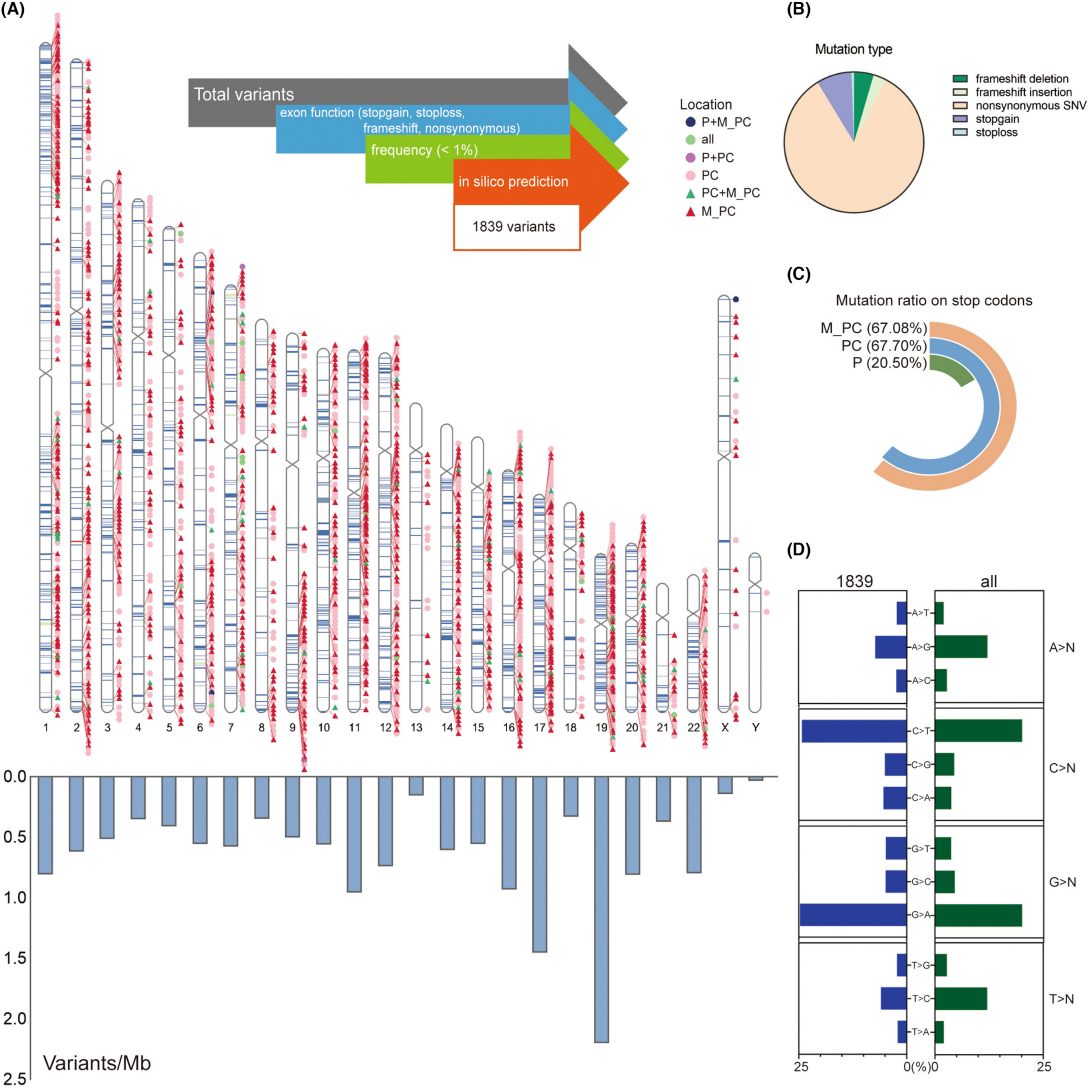
Workflow of filtering variants and variant analysis. (A) 1839 filtered variants were classified into six groups according to their sample groups and then displayed on the upper part of the chromosome map. The bar plot on the bottom showed variants per million bases on each chromosome. (B) Mutation types of 1839 variants include frameshift deletion, frameshift insertion, nonsynonymous single nucleotide variant (SNV), stop gain and stop loss. (C) Mutation ratios on stop codons in different groups. (D) Base substitution in 1839 variants and all detected variants. Each bar represented its proportion.

SNPs and InDels were filtered by limiting function, mutation type, frequency, and *in silico* prediction (Figure 1A). 1839 damaging mutations were identified within 1630 reference genes (Data S1), with the vast majority (83.74%) of them classified as nonsynonymous variants followed by stop gain (8.16%), frameshift deletion (4.57%), frameshift insertion (2.94%) and stop loss (0.60%) (Figure 1B). The mutation ratio of stop codons in M_PC and PC groups were almost the same, with 67.08% and 67.70% in contrast to 20.50% in the control group (Figure 1C). In addition, the mutations of C>T and G>A were predominant compared with others (Figure 1D). Chromosome 1 (Chr1) harboured the most damaging mutations (201 out of 1839), especially at 1p36. We considered the unequal length of each chromosome to better reveal the distribution of mutations and found Chr19 had the most mutation density (Figure 1A). The regions of major mutations occurred only in M_PC group were at 1p31‐p36, 1q32, 19q13, 19p13, 11p11‐p15, 11q11‐q14, while in PC group were 1p34‐p36, 11p11‐p15, 11q12‐q14, 17p11‐p13, 17q21‐q25, 19p13, 19q13.

In alignment with the previous findings,^10^ *TTN* and *PLEC* harboured the most damaging mutations in our study. *TTN* consists of 364 exons, transcribes an mRNA over 100 kb long, and encodes the largest known protein titin, which acts in critical structural, developmental, mechanical, and regulatory roles in cardiac and skeletal muscles.^28^ Besides, this gene owned a high correlation coefficient between *TTN*‐tumour mutation burden and tumour mutation burden‐WES for PCa.^29^ *PLEC*, a member of the Plakin family of proteins and a cytoskeletal linker, modulates protein kinase C signalling and mitogen‐activated protein (MAP) kinases involved in cellular stress responses and migration in cancer. High levels of PLEC are associated with PCa and metastasis.^30^

Some PCa genes were also identified as having damaging mutations, such as *FOXA1*, *NCOA1*, *BRCA1*, *EPHA6*, *SPOP*, etc. (Data S1). As a pioneer transcription factor, *FOXA1* targets the androgen receptor signalling pathway and acts as an oncogene in PCa.^31^ The mutation rate of *FOXA1* was much higher in the Chinese cohort (41%) than in TCGA PCa (4%).^31^ The pace was slightly higher in metastatic PCa (12%–13%) than in primary PCa (8%–9%) in other cohorts.^10^ Four frameshift deletions (Chr14: 38061131‐38061177) in *FOXA1* were identified in a single M_PC patient. One mutation in *CD34* (rs28362497:C>A) was identified in the M_PC group. *CD34* is observed as one of the hallmarks indicating tumour progression and metastasis.^32^ Increased expression of *CD34* confers tumour progression and aggressiveness on PCa accompanied by higher prostate‐specific antigen level, Gleason score, and the possibility of tumour recurrence.^33^

### 3.2 Transcriptomic architectures of PCa and metastasis

**FIGURE 2:**
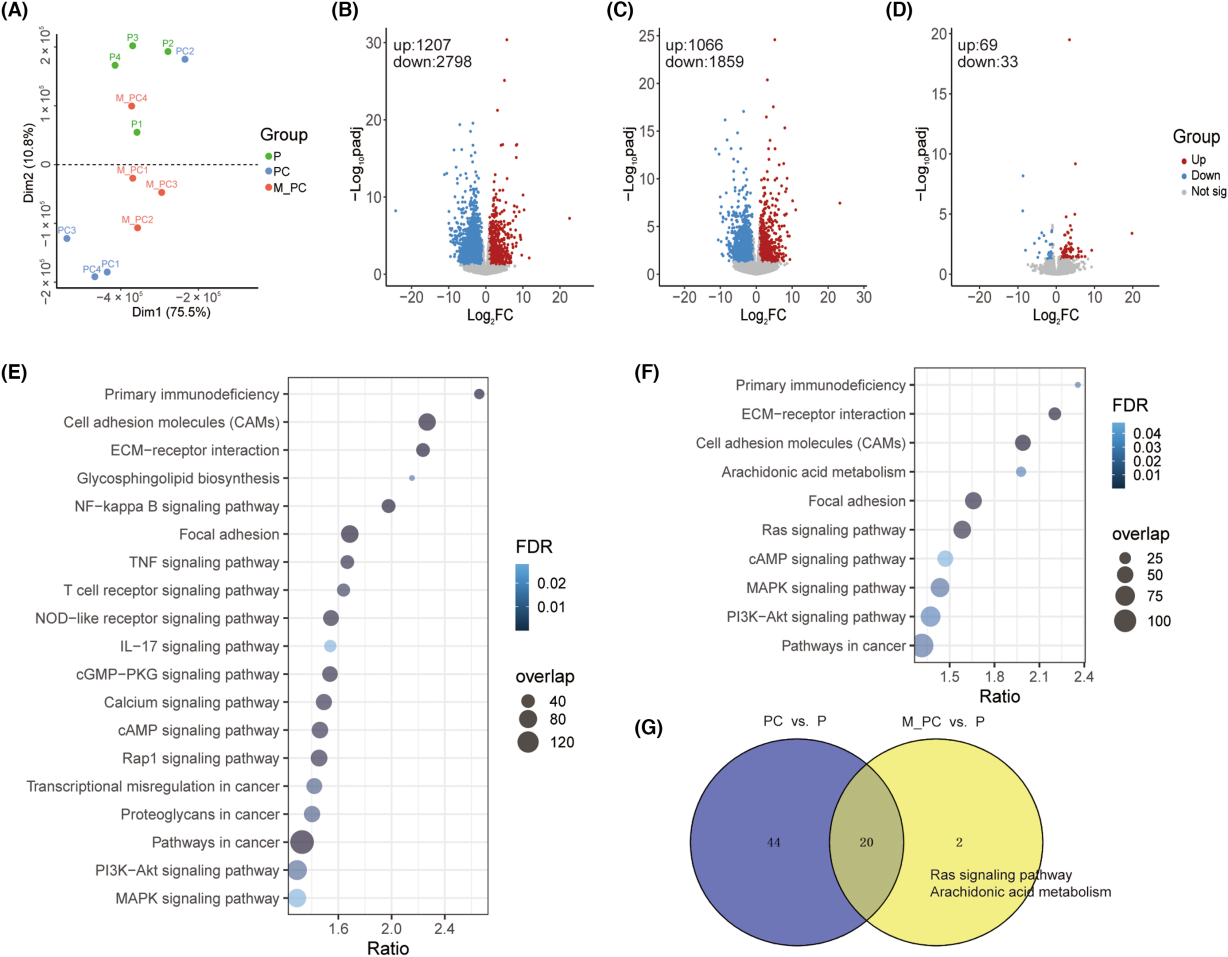
The expression differences in the two cancer groups compared to the control group. (A) PCA plot of sample expression profiles. Green dots: P, blue dots: PC, red dots: M_PC. (B–D) Volcano plot of differentially expressed genes (DEGs) between two groups. B: PC versus P, C: M_PC versus P, D: M_PC versus PC. Red dots represented up‐regulated genes (log_2_FC >1, padj <0.05). Blue dots represented downregulated genes (log_2_FC <−1, padj <0.05). The number of up‐and‐down‐regulated genes was shown in the upper‐left corner. (E, F) Bubble maps of pathway analysis for the DEGs. *X*‐axis: the enrichment ratio of DEGs. *Y*‐axis: the name of enriched pathways. The size of the node represented the number of enriched differential genes. The *p* value was represented by a colour scale, suggesting statistical significance. E: PC versus P, F: M_PC versus P. (G) Venn plot of pathways in each group. Two specific pathways in M_PC versus P were shown on the right.

We employed PCA and found the samples were clustered into three groups except for two outliers, PC2 and M_PC4 (Figure 2A), which were then removed for subsequent analysis. DEGs (adjusted *p* < 0.05 and |log_2_FC| > 1) were then identified using the DESeq2 package, with a total of 4005, 2925, and 102 yielded from each comparison (Data S2). Of these, 1207 genes were up‐regulated and 2798 genes were down‐regulated in the PC group compared to the P group (Figure 2B); 1066 genes were up‐regulated and 1859 genes were down‐regulated in the M_PC group compared to the P group (Figure 2C); accordingly, 69 genes were up‐regulated and 33 genes were down‐regulated in the M_PC group compared to the PC group (Figure 2D).

The over‐representation analysis was used to estimate the functions of the DEGs. With the threshold of FDR <0.05, 64 terms were significantly over‐represented for the DEGs identified between the PC and P groups, and 22 terms were enriched for the DEGs between the M_PC and P groups. However, no statistically significant term was enriched in M_PC and PC groups. Among these terms, several PCa‐related pathways were enriched (Figure 2E,F), including “pathways in cancer”, “ECM‐receptor interaction”, “cell adhesion molecules”, “focal adhesion”, “cAMP signaling pathway”, “MAPK signaling pathway”, and “PI3K‐Akt signaling pathway”. Additionally, 20 terms commonly occurred in both comparisons (PC vs. P and M_PC vs. P), indicating the similarity between PC and M_PC to some extent. Notably, two pathways, Ras signalling pathway and arachidonic acid (A.A.) metabolism, were exclusively presented in M_PC versus P group (Figure 2G). Studies have demonstrated Ras signalling participated in the process of metastasis by activating NF‐κB and MAPK members.^34^ A.A. induces bone marrow adipocyte differentiation and stimulates PC3 (one kind of human prostate cancer cell lines) invasion, causing bone metastasis observed in bone marrow co‐culture PC3 and bone marrow adipocyte.^35^

### 3.3 Gene expression clustering of PCa and metastasis

**FIGURE 3:**
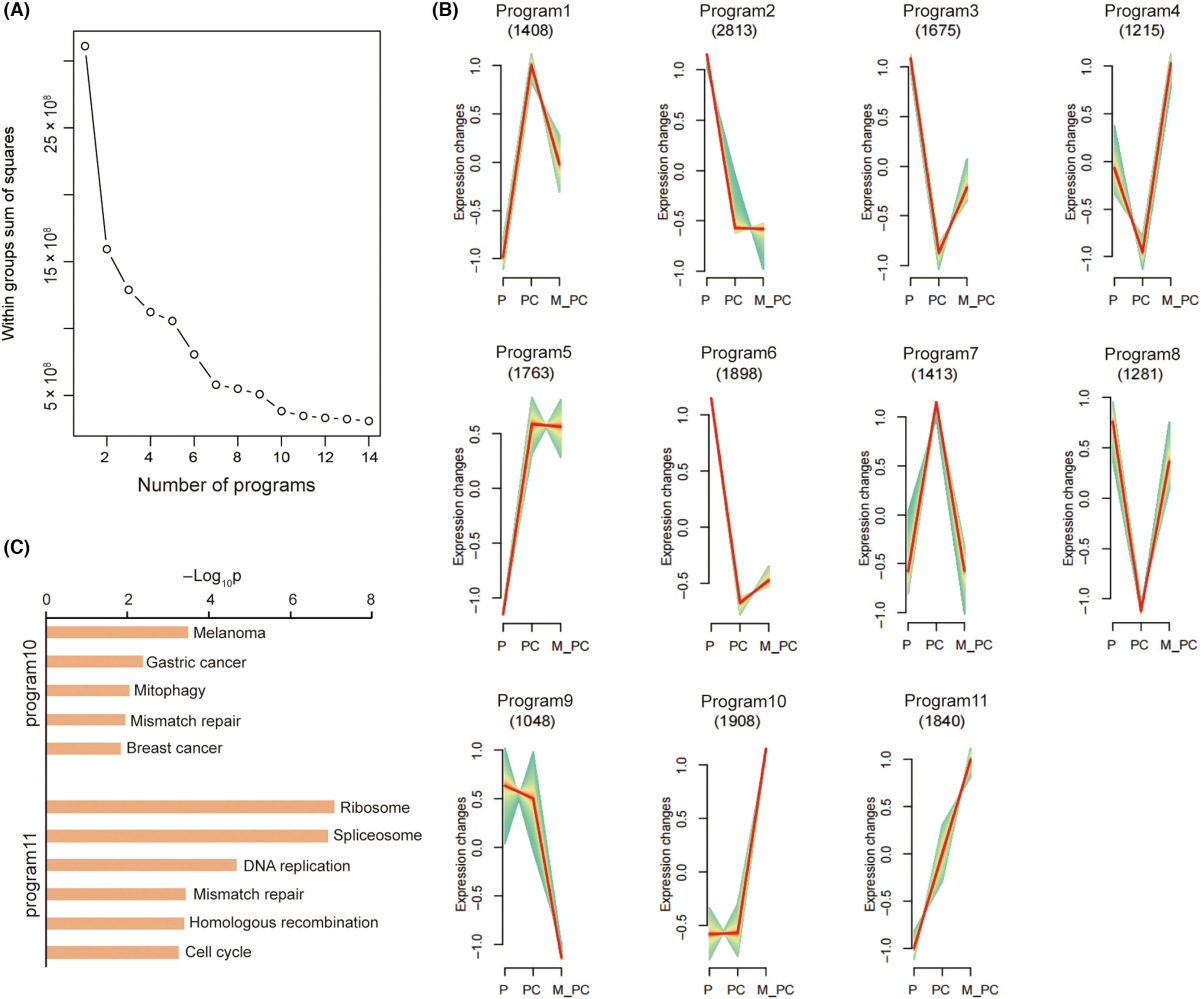
Clustering analysis and pathway analysis of two programmes. (A) Line chart of the result of K‐means. The number of collections was set as 11 for analysis. (B) Different gene expression patterns in three groups. Three groups on *X*‐axis from left to right were P, PC, and M_PC. The red line indicates the centre line of the average expression in each program. The number of genes in each program was shown at the top. (C) Pathway analysis of programs 10 and 11. The bar plot presented the enrichment scores (−log_10_p) of cancer‐related terms in the top 10 significantly enriched terms.

We next performed gene clustering analysis to identify the signature genes associated with metastasis. By applying the K‐means method, a total of 11 programmes were generated (Figure 3A,B), in which the gene numbers varied from 1048 to 2813. According to the trend of each cluster, programs 10 and 11 showed signatures that could better indicate the occurrence of metastasis.

In program 10, 5 KEGG pathways, including mismatch repair, mitophagy, melanoma, gastric cancer, and breast cancer, were enriched (Figure 3C). Genes related to three other cancers (melanoma, gastric, and breast cancer) suggested the potential function in PCa. Additionally, *SLC7A11*, a core gene of ferroptosis,^36^ was found in program 3. Ferroptosis promotes tumour metastasis or growth by driving the polarization of macrophages in the tumour microenvironment. The over‐expression of *SLC7A11* down‐regulates the sensitivity for ferroptosis execution and was an adverse factor for the disease‐specific survival of patients with PCa. The tumour mutation burden and neoantigen level are positively associated with *SLC7A11* expression.^37^

Mismatch repair recurred in program 11 (Figure 3C). Also, the analysis identified part of the genes enriched in the ribosome. Ribosomal proteins were over‐expressed in the prostate and could promote tumorigenesis by interactions with the *p53* tumour suppressor pathway and by direct effects on various oncogenes.^38^ Within genes enriched in the ribosome, *RPS21* exhibited positive relation with Gleason grade and participated in PCa cell proliferation and invasion.^39^

### 3.4 Signature genes associated with the PCa metastasis

**FIGURE 4:**
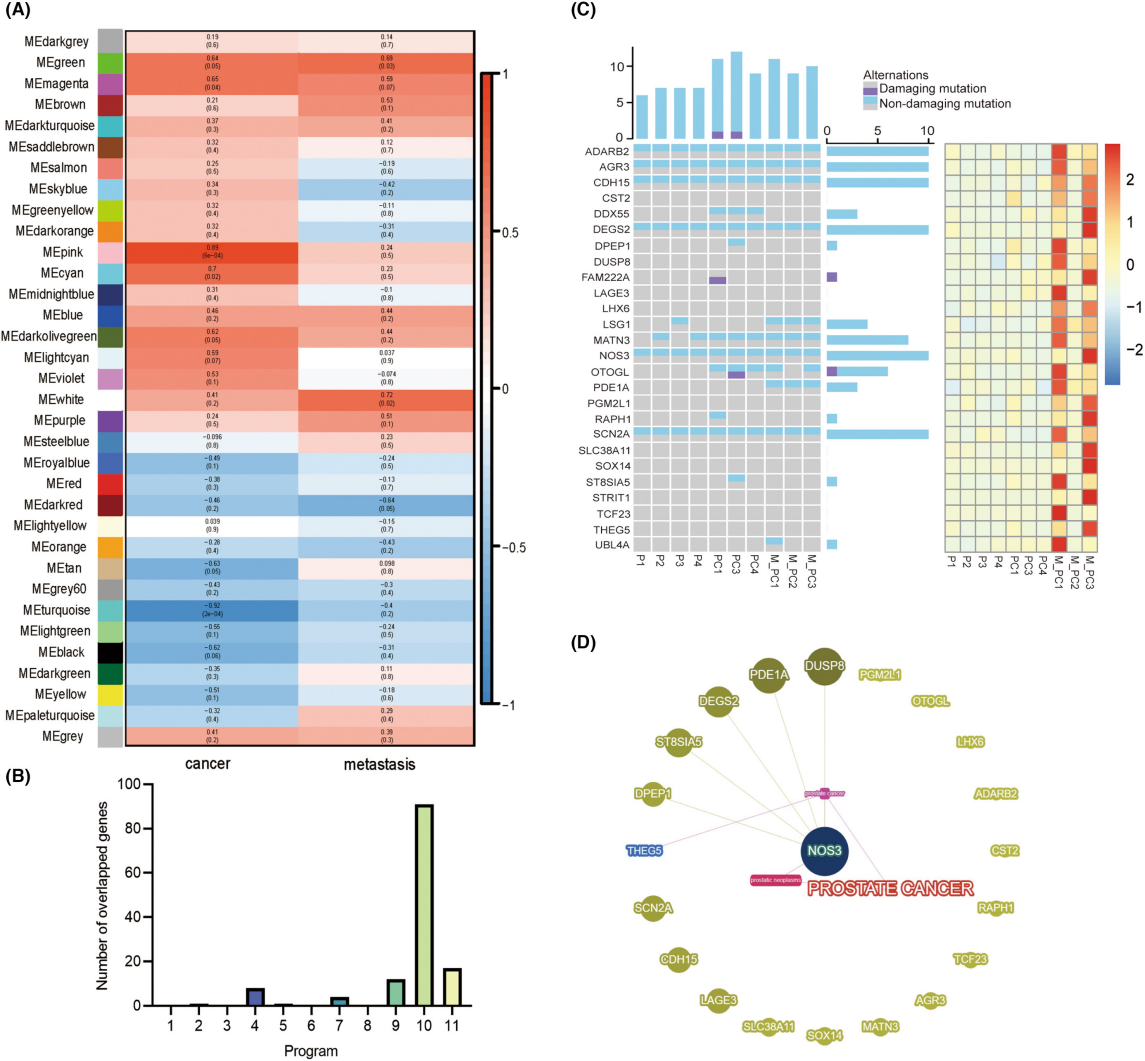
Identification of crucial modules and alterations of selected genes. (A) Correlation analysis of module eigengenes to metastasis. Each cell showed a correlation coefficient and corresponding *p* value. (B) The number of overlapped genes in each program compared to the key module. Each bar represented the number of overlapped genes in a different group, among which the number of program 10 was the max. (C) Alterations of 26 selected genes on exome and transcriptional levels. Left: Alterations of each gene in different samples. The top and right of the heatmap represented the number of genes mutated in one sample and the number of samples where one gene mutated. Right: Heatmap of 26 genes based on transcriptome data. (D) Network of genes from 26 genes and association with prostate cancer (PCa) and metastasis.

It has been widely recognized that co‐expressed genes are commonly involved in similar biological pathways or processes. Therefore, we constructed gene co‐expression networks using WGCNA to identify the genes that played a similar role in the process of metastasis. A total of 18,262 genes were parsed into 34 co‐expression modules. Test of associations between the metastasis state and the module eigengenes was performed and the white‐coloured module was shown to have the most positive association with the metastasis (*r* = 0.72; *p* = 0.02, Figure 4A).

The module harboured 135 genes. We further looked up the distributions of those genes in the 11 gene expression clusters. The result demonstrated 67.41% genes reoccurred in the program 10 (Figure 4B). Of them, 26 genes were further retained, as their expression levels showed significant differences between the M_PC and P groups. Among the 26 genes, 18 genes showed significant expression differences between the M_PC and PC groups (Table S2). Their exomic and transcriptomic profiles were shown in Figure 4C. It is worth noting that these genes were specifically highly expressed in M_PC, and only two genes contained damaging mutations (FAM222A: rs199694375; OTOGL: rs78377084, Chr12: 80747138, C>T).

We further explored the 26 genes under the specific phenotypes and diseases (keywords: prostate cancer and metastasis) in the Phenolyzer (https://phenolyzer.wglab.org)^40^ (Figure 4D) and found *NOS3* played a relatively crucial role in these selected genes. *NOS3* (endothelial nitric oxide synthase or *eNOS*) was highly expressed in metastatic PCa and was targeted by microRNA to suppress bone metastasis.^41^ In addition, other genes have also been associated with cancer metastasis. *CST2* and *PGM2L1* functioned in the cell migration and invasion of PCa.^42^, ^43^ The function of *AGR3* in cancer development remains unclear. However, AGR3 could bind to metastasis‐related proteins C4.4a and DAG‐1, and co‐expression analysis depicted some AGR3 partners linking AGR3 with cell adhesion, tight junction, motility, metastasis, and regulation of cell cycle.^44^ *RAPH1*, *SOX14*, *DPEP1,* and *UBL4A* have a significant role in the invasion or metastasis of breast cancer, cervical cancer, colon cancer, and pancreatic ductal adenocarcinoma, respectively.^45^, ^46^, ^47^ To confirm any transcription factors for the 26 genes, we searched in Cistrome Data Browser and discovered *LHX6*, a tumour suppressor gene, as a factor in colorectal adenocarcinoma.^48^ *LHX6* was observed down‐regulated in PCa cell lines^49^ while significantly up‐regulated between M_PC and P group (log_2_FC = 1.79, padj = 0.04).

### 3.5 Landscape of mutations and expression level changes for essential genes

Previous research has reported some essential molecules in PCa.^14^, ^50^ Therefore, we focused on 62 genes that have key roles in several aspects, including ETS, R.B., EPI, *AR* signalling pathway, PI3K signalling pathway, Wnt signalling pathway, and DNA repair (other unclassified genes were grouped into others).

**FIGURE 5:**
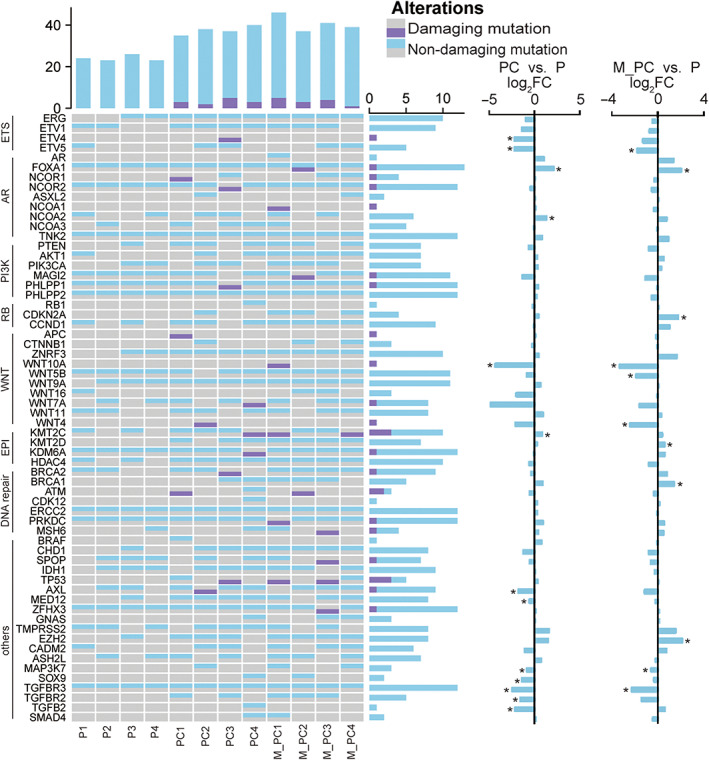
The landscape of alterations of crucial genes in prostate cancer (PCa). The top and the first bar graph on the right of the heatmap represented the number of genes mutated in one sample and the number of samples where one gene mutated. Two bar graphs on the right represented the differences in expression levels of critical genes between the two groups of cancer and the control group, respectively. *p < 0.05.

Only 21 genes harboured damaging mutations, with *TP53* and *KMT2C* having a higher mutation rate (Figure 5). Compared to *KMT2C*, there were no significant statistical differences between *TP53* in PC or M_PC. Six genes (*ETV4*, *FOXA1*, *WNT10A*, *WNT4*, *KMT2C* and *AXL*) were differentially expressed (padj < 0.05) in PC or M_PC. *ETV4*, *WNT10A,* and *AXL* were down‐regulated in PC, while *FOXA1* and *KMT2C* were up‐regulated in PC. In M_PC, *FOXA1* was over‐expressed, while *WNT10A* and *WNT4* were down‐regulated. *FOXA1*, with damaging mutations in M_PC2, was the sole one over‐expressed in PC and M_PC simultaneously. In contrast to *FOXA1*, *WNT10A* showed the most down‐regulation in both PC (log_2_FC = −4.29) and M_PC (log_2_FC = −3.32). On the other hand, 13 genes showed expression differences but without damaging mutations (Figure 5). *CDKN2A*, *KMT2D*, *BRCA1*, and *EZH2* were up‐regulated only in M_PC, while *WNT5B* was merely down‐expressed in M_PC. Four genes (*MED12*, *SOX9*, *TGFBR2,* and *TGFB2*) were down‐regulated in PC and three (*ETV5*, *MAP3K7,* and *TGFBR3*) were down‐expressed in both PC and M_PC. ETS family showed a downward trend no matter in PC or M_PC.

## 4 DISCUSSION

With the development of PCa, metastasis will eventually happen to patients, and metastases are the final result of a multi‐stage process. This process depends on the specific phenotypic characteristics of tumour cells and the interaction with the host microenvironment and immune system. Genetic alterations in primary and metastatic tumours may contribute to these phenotypes and host interactions.^3^ In this study, we conducted a comprehensive analysis by comparing patients without cancer, patients with primary cancer and metastatic cancer. Comparing the genetic similarities and differences between primary and metastatic tumours may provide new insights into the biology of metastasis.

With the assistance of WES, we discovered that some chromosomal regions were highly mutated. Chr1 had the highest frequency within the filtered 1839 variants. *WNT4* located in 1p36 and the induction of WNT4/TCF7L1 resulted in increased malignancy in prostate cancer that was linked to dysregulation of androgen receptor signalling and activation of the *IL‐8/CXCR2* pathway.^51^ Furthermore, several essential genes were located in highly mutated regions such as *CCND1* (11q13). *CCND1* was over‐expressed in more aggressive prostate cancer phenotypes, and its expression was regulated by oestrogens via ERβ and might contribute to the progression and pathogenesis of prostate cancer.^52^ Previous studies found that genes like *KMT2D* and *BRCA1* mutation frequency were higher in metastatic samples.^3^ Although non‐damaging mutations were identified in both *KMT2D* and *BRCA1*, their expressions were found significantly higher in M_PC. Similar situations happened to other vital genes such as *EZH2*, *CDKN2A*, and *ETV5*. From this perspective, we could see that the modifications of genes were not wholly consistent with previous studies. Although genes might have multiple forms of mutations in different types of samples, their expression differences would intuitively be reflected at the RNA level, which also showed the necessity and advantage of multi‐omics combination on the other hand.

Our pathway analysis for the DEGs revealed several common pathways between PC and M_PC, which have already been identified as participants in various cancers, such as the PI3K‐Akt signalling and MAPK signalling pathway. More importantly, we found two pathways, the A.A. metabolism pathway and Ras signalling pathway, were specifically enriched in the M_PC group. The A.A. metabolism pathway has been proven essential in developing and progressing malignant PCa. As a member of the A.A. pathway, *ALOX15* (*LOX15* or *15‐LO‐1*) was found highly expressed in M_PC and PC groups. One of the methylated CpG dinucleotides in the *ALOX15* promoter was correlated with microdissected high‐grade prostatic intraepithelial neoplasia, PCa and metastatic human prostate tissues indicating *ALOX15* was a valuable marker for disease initiation and progression.^53^ *GPX3* was clearly down‐regulated in PCa which was consistent with the previous result.^54^ The expression of *GPX3* was negatively associated with PCa progression and its overexpression inhibited the PCa invasiveness and the expression of c‐met.^54^ Ras signalling was involved in various cancers' metastasis. In PCa, the Ras signalling network activated NF‐κB and MAPK members in the process of metastasis.^34^ The Ras/Raf/MEK/ERK/Elk‐1 signalling cascade was the central backbone in regulating bone metastasis.^55^ Among those genes enriched in Ras signalling, several genes belong to fibroblast growth factors (FGF) and relevant receptors. FGF signalling mediates cell secretion to influence PCa metastasis.^56^

To clarify gene expression change in different groups, we performed clustering analysis, and part programmes presented high expression in M_PC. There was an upward tendency in program 11, and pathway analysis suggested that genes in program 11 were related to DNA replication and cell proliferation. Besides, we used WGCNA to combine phenotype and expression and connected with clustering results to narrow programmes. Twenty‐six genes were found by selecting the pattern and module that best matched the feature of metastasis. Among them, several genes had been identified to play a role in the progression and metastasis of PCa, and the remaining were associated with other cancers. *CST2* functioned in the migration and invasion of PCa cells with a crucial role in cell adhesion molecules, extracellular matrix‐receptor interaction and focal adhesion.^42^ *PGM2L1* was involved in multiple processes in PCa, including glucose consumption, lactic production, cell migration and invasion.^43^ *DPEP1* was prominently up‐regulated in colorectal cancer during the transition from low‐grade to high‐grade intraepithelial neoplasia and functioned as a positive regulator for metastasis by regulating E‐cadherin expression.^57^ While in pancreatic ductal adenocarcinoma, *DPEP1* was negatively associated with the histological grade and invasion of tumour cells.^58^ *LHX6* inhibits tumorigenesis and progression in multiple cancers by regulating various pathways or genes which were involved in proliferation, apoptosis, migration, and cell cycle.^59^ *LAGE3* promoted migration and invasion of hepatocellular carcinoma by facilitating the JNK and ERK signalling pathways.^60^ *UBL4A* inhibits metastasis of pancreatic ductal adenocarcinoma.^47^

## 5 CONCLUSIONS

In summary, our WES and RNA‐Seq analysis provides several novel signature genes and pathways that potentially involve in PCa metastasis. These findings extended our understanding of the molecular mechanisms underlying the metastasis of PCa and would be ultimately beneficial for future prevention, diagnosis, and treatment.

## AUTHOR CONTRIBUTIONS

**Chongjun Xiang:** Data curation (supporting); formal analysis (lead); software (equal); visualization (equal); writing – original draft (equal). **Yue Li:** Formal analysis (equal); writing – original draft (equal). **Wenting Wang:** Data curation (lead). **Huiying Tao:** Software (equal). **Ning Liang:** Visualization (equal). **Shuang Wu:** Resources (equal). **Tianxi Yu:** Resources (equal). **Xin Cui:** Resources (equal). **Yaqi Xie:** Resources (equal). **Hongwei Zuo:** Resources (equal). **Chunhua Lin:** Conceptualization (equal); funding acquisition (equal); project administration (lead); supervision (equal). **Fuyi Xu:** Conceptualization (equal); funding acquisition (equal); methodology (lead); project administration (supporting); software (equal); supervision (equal); writing – review and editing (lead).

## FUNDING INFORMATION

This research was funded by Taishan Scholar Program (grant number Tsqn202103198); Natural Science Foundation of Shandong Province (grant number ZR2019MH132); Yantai Key Research and Development Project (grant number 2020MSGY079 and 2019MSGY135); Binzhou Medical University Research Start‐up Fund (grant number 50012305190).

## CONFLICT OF INTEREST STATEMENT

The authors confirm that there are no conflicts of interest.

## DATA SUBMISSION

The sequencing data have been already submitted to Sequence Read Archive (SRA) of NCBI and were available at PRJNA883010.

## Supporting information


Table S1.

Table S2.

Data S1.

Data S2.
